# Prognostic Roles of Blood Inflammatory Markers in Hepatocellular Carcinoma Patients Taking Sorafenib. A Systematic Review and Meta-Analysis

**DOI:** 10.3389/fonc.2019.01557

**Published:** 2020-01-29

**Authors:** Lixing Liu, Yang Gong, Qinglin Zhang, Panpan Cai, Li Feng

**Affiliations:** ^1^Department of Chinese Medicine, National Cancer Center, National Clinical Research Center for Cancer/Cancer Hospital, Chinese Academy of Medical Sciences and Peking Union Medical College, Beijing, China; ^2^The General Hospital of Shenyang Military Region, Shenyang, China

**Keywords:** neutrophil-to-lymphocyte ratio, platelet-to-lymphocyte ratio, hepatocellular carcinoma, sorafenib, inflammatory biomarkers

## Abstract

**Objective:** The purpose of this meta-analysis is to investigate the effectiveness of the prognostic roles of blood inflammatory markers in hepatocellular carcinoma (HCC) patients receiving sorafenib.

**Methods:** We carried out a comprehensive literature search in four databases. Study endpoints, hazard ratios (HRs) and the associated 95% confidence intervals (CI) for clinical outcomes, which were to assess therapeutic efficacy, were extracted. This meta-analysis was conducted by Review Manager 5.3.

**Results:** We summarized the available evidence from 18 studies with a total of 2,745 cases. The pooled results showed that the synthesized HR favored patients with low pretreatment NLR (neutrophil-to-lymphocyte ratio), which also indicated that HCC patients with a lower baseline NLR may have a better response to sorafenib than those with higher NLR (HR = 1.76, 95% CI [1.44, 2.15], *P* < 0.00001, *I*^2^ = 68%). Significance was also observed for the prognostic function of the PLR (platelet-to-lymphocyte ratio) of HCC patients treated with sorafenib (HR = 1.49, 95% CI [1.16, 1.93], *P* = 0.002, *I*^2^ = 0%, *P* = 0.65). The subgroup analysis revealed that different gene backgrounds play a prominent role in the source of heterogeneity. Interestingly, the predictive effect on OS (overall survival) was more pronounced as the NLR cutoff value increased. Notably, a significant predictive effect of NLR on the clinical outcome was detected in HCC patients treated with sorafenib compared to those treated with tivantinib.

**Conclusion:** In conclusion, the present study reported promising predictive biomarkers for HCC patients and notably indicated that HCC patients with a lower baseline NLR and PLR may have a better response to sorafenib than those with higher ones. Further large-scale prospective studies are required to determine the optimal NLR and PLR cutoff values, which are important for identifying the dominant populations for sorafenib treatment.

## Introduction

According to GLOBCAN 2018, there were 841,080 estimated new liver cancer cases worldwide in 2018 (1). The global five-year survival rate of hepatocellular carcinoma (HCC) is between 5 and 30% (2). Sorafenib is one of the two approved first-line molecular-targeted drugs for the treatment of advanced HCC that has brought great hope to patients. Molecular-targeted medicine can regulate the signal transduction of cell proliferation, angiogenesis, and tumor suppressor gene loss, thereby achieving the purpose of inhibiting the growth and proliferation of tumor cells, which is very promising for cancer treatment. However, the unconfirmed effectiveness for a single patient countered with the heavy economic burden and possible serious side effects make a number of patients hesitant to choose this therapy, especially in low-income countries. Therefore, elucidating the dominant population that responds to different treatments could allow patients to get the fullest benefits and reduce economic waste, which are of great significance to patients, families, and society as a whole. However, there is lack of prognostic biomarkers for HCC patients to effectively predict the outcomes and benefits from sorafenib. Therefore, development of more convenient and effective indicators to help clinicians and patients understand the prognosis and benefits of treatment programs is needed.

The inflammatory and immune microenvironments play crucial roles in each stage of cancer formation and progression. White blood cells, particularly lymphocytes, as reflections of the inflammatory response in the tumor immune microenvironment have been reported as effective predictors of cancer progression (3–5). Emerging studies have shown that the neutrophil-to-lymphocyte (NLR) and platelet-to-lymphocyte (PLR) could predict outcomes for HCC patients (6–9). However, there is no consensus on whether they have the same prognostic role for HCC patients treated with sorafenib (10, 11). Thus, we conducted this meta-analysis to investigate the effectiveness of the prognostic roles of inflammatory markers in HCC patients receiving sorafenib based on the current evidence.

## Methods

### Search Strategy

We carried out a related topic search of this meta study in four databases (PubMed, Cochrane Library, Embase, and Web of Science) with the following MeSH terms and keywords: “hepatocellular carcinoma,” “inflammatory markers,” “neutrophil,” “lymphocyte,” “platelet,” “neutrophil to lymphocyte ratio,” “platelet to lymphocyte ratio,” “molecular targeted therapy,” “sorafenib,” and “prognostic factors.” The specific search strategies for the four databases are given in the Supplemental maTerials (Tables S1–S4). There was no language restriction in the literature search. The references of related literature and reviews were also manually searched to identify additional eligible studies. The protocol for this meta-study was reviewed and registered on PROSPERO (CRD42019120884).

### Inclusion and Exclusion Criteria

This meta-analysis included cohort studies to observe the association between baseline NLR, PLR, or other inflammatory factors and survival outcomes in patients with HCC treated with sorafenib, aiming to explore the predictors of the efficacy of sorafenib.

The inclusion criteria for each study were as follows: (1) Cohort studies involved the patients with HCC receiving sorafenib or molecular targeted therapies; (2) articles analyzing the correlation between inflammatory markers and survivals of HCC patients including studies that investigated and reported the predictive effect of the NLR or PLR; (3) Studies must have included a cutoff value of the peripheral blood inflammatory markers with a comparison between different groups according to the cutoff value. Data regarding overall survival (OS), progression free survival (PFS) or other clinical outcomes, related hazard ratio (HR), and 95% confidence interval (CI) must also have been available; and (4) the publication was in English and access to the full article was required. The exclusions of this research were insufficient data, case report, conference abstract, comments, and duplicate publications.

### Data Extraction (Selection and Coding)

Two reviewers (LX Liu, QL Zhang) worked independently to screen the studies according to the inclusion and exclusion criteria. The basic information (study design, first author, published year, HCC stage, sample size, inclusion date, average age, and study location) of the included studies together with the cut-off value of inflammatory markers, treatment, follow-up time, study endpoints, and available data to assess the therapeutic efficacy were also extracted. If these data were not available, the HR or 95% CI was estimated by Review Manager 5.3 software according to its *P*-value. If the survival curves were the only useful data in the articles, Peto's method was applied to extract the HR value and CI (12). Different opinions were discussed and a third reviewer (L Feng) would join in to reach consensus. The data extraction procedure followed the rules of PRISMA.

### Risk of Bias (Quality) Assessment

The qualities of the included studies were assessed by two independent researchers using the Newcastle-Ottawa Quality Assessment Scale (NOS) (13–15). Studies were assessed by two reviewers (LX Liu, QL Zhang) by the following aspects: (1) representativeness of the exposed cohort; (2) selection of the non-exposed cohort; (3) ascertainment of exposure; (4) demonstration that outcome of interest was not present at the start of the study; (5) study controls for factors like age; (6) study controls for any additional factors; (7) assessment of outcomes; (8) long enough follow-up; and (9) adequacy of follow up of cohorts. High quality studies were defined as having a score of ≥ 7. If there was any disagreement, a third reviewer (L Feng) would be available to discuss and resolve the different opinions.

### Strategy for Data Synthesis

All data were analyzed to perform the meta-analysis using the software of Review Manager 5.3. The effects were measured using a HR and Outcome data were weighted by generic inverse variance. The HRs and 95% CIs were analyzed to investigate the relationship between inflammatory markers and clinical outcomes of HCC patients. A chi-squared test was used to evaluate the heterogeneity of therapeutic efficacy in the trial when *P* < 0.1 was significant. The levels of heterogeneity were assessed by the *I*^2^ statistic (*I*^2^ > 75% considerable heterogeneity; *I*^2^ > 30% moderate heterogeneity). Studies with high level heterogeneity were analyzed by a random-effect model (*I*^2^ > 25%). Otherwise, a fixed-effect was employed. The subgroup analysis or sensitive analysis was performed when there was significant clinical or statistical heterogeneity. We analyzed quality of trials and excluded studies at high risk of bias. Publication bias was signified by Funnel plots. Because included studies were evaluated in various populations in different countries and with different cutoff values of biomarkers or with different research quality, we conducted subgroup analyses to minimize the impacts of the different conditions. A two-sided *P* < 0.05 was considered statistically significant.

## Results

### Characteristics of Included Articles

**Figure 1:**
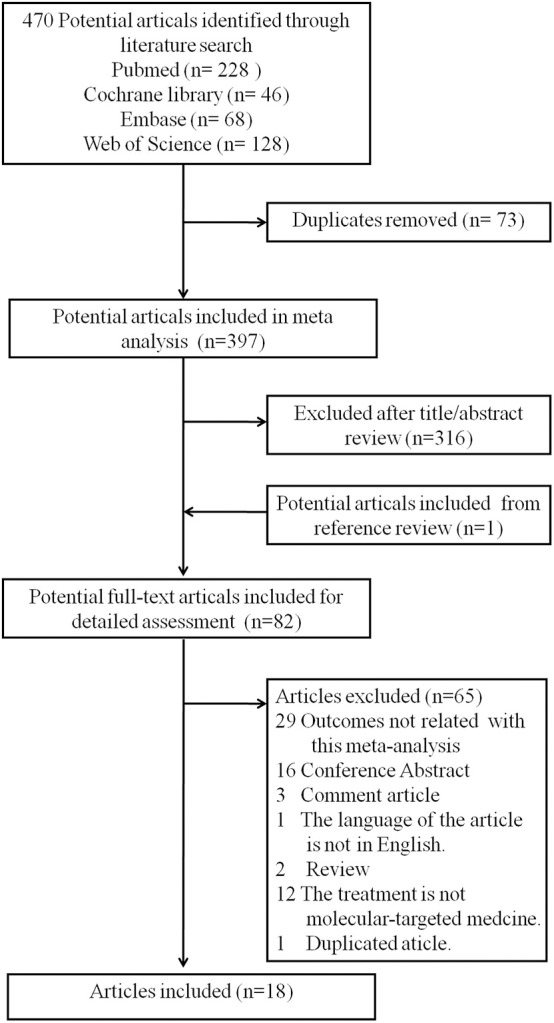
Flow diagram of the literature search and studies inclusion.

A total of 470 potential articles were identified through the literature search [PubMed (*n* = 228), Cochrane library (*n* = 46), Embase (*n* = 68), and Web of Science (*n* = 128), Tables S1–S4]. Overall, 73 duplicates studies were removed, 316 articles were excluded after title/abstract review, and one potential article was added from the reference review. A total of 82 potential full-text articles were included for detailed assessment. Then, after carefully reviewing the full text, 65 articles were excluded as follows: 29 studies with outcomes not related to this meta-analysis, 16 conference abstracts, 3 comment articles, one article in Japanese, 2 reviews, 12 studies including patients without molecular-targeted medicine treatment, and one duplicated article (Table S5). Finally, 18 studies (16–33) that encompassed 2,745 patients with advanced HCC were included according to the inclusion criteria. A flowchart recorded the eligible study selection (Figure 1) and the main information of the studies included in this analysis are presented in Table 1.

**Table 1:** Baseline characteristics of the included studies, inflammatory indicators, primary end points, and quality of the study according to the NOS scale.

**References**	**Origin**	**Study design**	**Stage of HCC**	**Treatment**	**Inclusion period**	**Age (years)**	**Follow up period (months)**	**Sample size**	**Biomarker**	**Cutoff value/Time of assessment**	**Study endpoints**	**Quality score**
Zheng et al. (27)	China	Retrospective	Advanced	Sorafenib	01/2011– 12/2012	55 ± 12	8.9 (2–25.5)	65	NLR	4/ baseline	OS/TTP	9
da Fonseca et al. (20)	Brazil	Retrospective	Advanced	Sorafenib	07/2009– 11/2013	59.7 (19–80.3)	11.4 (0.47–27.1)	105	NLR	3.5/7 days before treatment	OS	8
Wei et al. (24)	China	Retrospective	Intermediate-advanced	TAE combined with Sorafenib	01/2010– 05/2013	58.7 ± 10.1	16.5 (8–38)	40	NLR	3/baseline	OS	9
Diaz-Beveridge et al. (19)	Spain	Retrospective	Advanced	Sorafenib	01/2008– 04/2015	62 (26–82)	Median:43	145	NLR	4/baseline	OS	8
Zhang et al. (26)	China	Retrospective	Stage II/III	Curative resection adjuvant sorafenib	08/2009– 03/2012	Median:51	Median:28.6	38	NLR	Change of NLR	OS/RFS	9
Luè et al. (22)	Spain	Retrospective	Intermediate-advanced	Sorafenib	08/2005– 10/2013	63 ± 11 (28–86)	7 (3–15)	154	NLR	2.3/baseline	OS	8
Personeni et al. (23)	Europe and The North America	Prospective RCT	Advanced	Tivantinib/placebo	10/2009– 08/2011	Tivantinib 240 mg 69(45–83); 360 mg 71 (27–80); Placebo 68 (46–85)	18.9 (0.6–24.8)	98	NLR	3.0/baseline	OS/TTP	8
Yuan et al. (25)	China	Retrospective	Advanced	Sorafenib	07/2008– 12/2012	52.5 (21–78)	8.5 (1.0–80.5)	120	Neutrophils count	3.65 × 10^9^ neutrophils/L/1 week prior to treatment	OS	9
Bruix et al. (17)	Europe, America, Australia and Asia	Retrospective	Advanced	Sorafenib/placebo	03/2005– 07/2007	64 (21–89)	NR	827	NLR	3.1/baseline	OS	9
Howell et al. (21)	Japan, Italy and UK	Prospective	Advanced	Sorafenib	01/2005– 12/2015	70 ± 10	7.1 (3.4–16.1)	442	NLR	2.52/baseline	OS	9
Afshar et al. (16)	UK	Retrospective	Advanced or metastatic	Sorafenib	04/2009– 03/2014	66 (60, 74)	NR	231	NLR	3/baseline	OS	7
Zhu et al. (28)	China	Retrospective	Advanced	Sorafenib with or without TACE	01/2013– 12/2016	54.29 (24–86)	Every 8 weeks	142	MLR	0.35/baseline	OS/PFS	8
Casadei Gardini et al. (18)	Italy	Retrospective	Advanced HCC	Sorafenib	2012– 2015	NR	NR	56	NLR	3/ baseline	OS/PFS	7
Miyahara et al. (29)	Japan	Retrospective	Advanced HCC	Sorafenib	07/2009– 10/2010	71.5 (36–84)	Every month	30	Ang-2, G-CSF, HGF, and leptin	baseline	PFS	9
Katayama et al. (30)	Japan	Retrospective	Advanced HCC	Sorafenib	01/2009– 10/2015	67.1 ± 11.3	NR	17	NLR	2/baseline	TTUP	8
Goyal et al. (31)	America	Prospective	Advanced HCC	Sorafenib+mFOLFOX treatment	01/2013– 05/2017	65 (29–76)	Median:10.7	40	IL10, IL8, sMET, CD56 Bright NK lymphocyte	baseline	OS/TTP	8
Cho et al. (32)	Korea	Retrospective	Advanced HCC	Sorafenib	10/2008– 11/2012	52.41 ± 8.67 (38–73)	7 (1–44)	34	IL-17A	1.94 pg/mL/baseline	OS/PFS	8
Conroy et al. (33)	France	Retrospective	Advanced HCC	Sorafenib	10/2007– 09/2015	67.1 (44.6–80.1)	11.74 (0.2–73.0)	161	NLR/PLR/LMR	4/200/3/baseline	OS	7

*TTUP, Time to untreatable progression*.

Generally, the 18 included studies were published from 2011 to 2018. Three of them were prospective studies (21, 23, 31) and 16 were retrospective studies. The 194 patients in two studies (22, 24) had intermediate-advanced HCC, and 2,551 patients in the remaining studies were in the advanced stage of the disease. Eight studies were conducted in Asia, mainly in China and Japan, 8 studies were conducted in America and Europe, and two were multicentered covering locations in western and eastern countries (17, 21). Thirteen studies focused on the NLR as prognostic factors, one study analyzed the association between neutrophil count and survival outcome (25), three studies explored the PLR prediction function, one study investigated the prognostic function of the monocyte -to-lymphocyte ratio (MLR) (28) and one the lymphocyte-to-monocyte ratio (LMR) (33), and three studies reported on inflammation biomarkers as risk factors for survival, such as IL-17A, IL8, IL10, Ang-2, G-CSF, HGF, and leptin, etc.

### Quality (Risk of Bias) Assessment

We evaluated the included studies according to the NOS: 7 studies had a score of 9, 8 had a score of 8, and 3 had a score of 7. The details of assessment results are presented in the Table S6.

### NLR and OS

**Figure 2:**
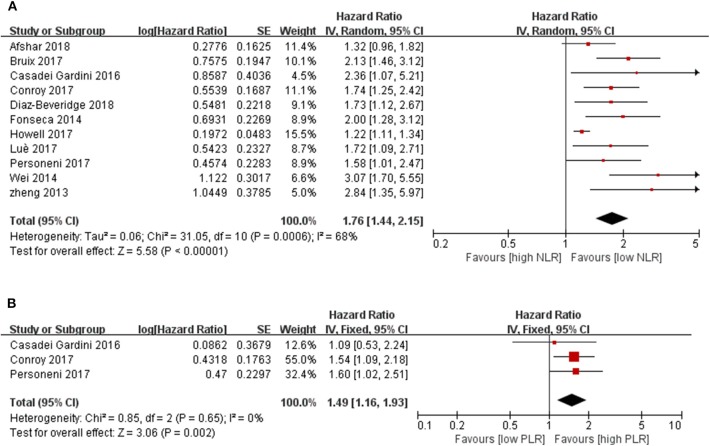
Forest plots for associations between pretreatment **(A)** blood NLR and survival, **(B)** blood PLR and survival.

All included studies were adjusted for potential confounders applying the COX proportion hazard model. Among the 13 studies focused on the NLR as a prognostic factor, a significant relationship between increased NLR after sorafenib therapy and survival outcome was reported in Zhang's study (HR = 4.647, 95 % CI 1.266–17.053, *P* = 0.021) (26). Katayama found that a low NLR was a useful predictor of time to untreatable progression in 17 advanced HCC patients treated with sorafenib for more than 12 months [time to tumor progression (TTP), HR:0. 271, 95 % CI 0.074–0.997, *p* = 0.050] (30). Thus, the relationship between pretreatment NLR and OS were investigated in 11 studies with 2,324 cases in the final meta-analysis, and a close relationship was detected (*P* < 0.00001). The pooled results showed that the synthesized HR favored patients with low pretreatment NLR, which meant that a lower baseline NLR in HCC patients correlated with significantly better OS, which also indicated that HCC patients with a lower pretreatment NLR may have a better response to molecular targeted medicine than those with higher NLR (HR = 1.76, 95% CI [1.44, 2.15], *P* < 0.00001, *I*^2^ = 68%, Figure 2A).

### Subgroup Analysis

Due to the high *I*^2^ values in the pooled analysis, subgroup analyses were also performed. We conducted the subgroup analyses following several related clinical parameters as possible sources of heterogeneity and the results are summarized in Table 2. Interestingly, the subgroup analyses showed marked change by the clinical parameters. In the subgroup analysis of study design and cutoff values, the results showed an obvious decrease in heterogeneity (Table 2). Of note, in the research region subgroup, the heterogeneity reduced to zero [Asia group: 2.98 (1.88, 4.73), *P* < 0.00001, *I*^2^ = 0%, *P* = 0.87; Europe and America group: 1.65 (1.41, 1.93), *P* < 0.00001, *I*^2^ = 0%, *P* = 0.73, Table 2]. However, the multicenter studies involving countries from Europe, America, and Asia showed a high heterogeneity and revealed little OS prediction function of NLR [1.56 (0.90, 2.69), *P* = 0.11, *I*^2^ = 87%, *P* = 0.005, Table 2]. This indicated that different genetic backgrounds played a prominent role in the source of heterogeneity. These two multicenter studies involved 827 and 442 cases, respectively, which accounted for a large proportion of the entire meta-analysis. The other subgroup analyses with statistically significant heterogeneity all involved the two studies, such as subgroup of sample size, age, follow-up time, and study quality, and the combined weights of the two studies in each subgroup analysis were 36.3, 38.0, 30.7, and 32.6%, respectively. Another interesting finding in the subgroup analysis was that the predictive effect for OS was more pronounced as the NLR cutoff value increased. A significant outcome prediction relationship was seen in the study with an NLR cutoff value of ≥ 3 but not in group with NLR ≥ 2 [2 ≤ NLR < 3: 1.34 (0.99, 1.82), *P* = 0.06, *I*^2^ = 53%, *P* = 0.15, Table 2]. A significant predictive effect of NLR on the clinical outcome was detected in HCC patients treated with sorafenib [1.69 (1.38, 2.08), *P* < 0.00001, *I*^2^ = 66%, *P* = 0.003, Table 2]. However, marginally statistical significance was found in HCC patients following tivantinib treatment [1.58 (1.01, 2.47), *P* = 0.05, Table 2].

**Table 2:** Subgroup analysis of the correlation between inflammatory index and survival outcome was was conducted according to different influencing factors and parameters, such as study design, research area, sample size, age, inflammatory index cut off value, and follow-up time, etc.

**Variables**		**No of studies**	**No of patients**	**Test of association**			**Test of heterogeneity**	
				**HR**	**95% CI**	***P*-value**	***I*^**2**^ (%)**	***P*-value**
**Overall Survival**								
	Total	11	2,324	1.76	[1.44, 2.15]	*P* < 0.00001	68	*P* = 0.0006
**Study Design**								
	Prospective	2	540	1.23	[1.12, 1.35]	*P* < 0.00001	20	*P =* 0.26
	Retrospective	9	1,784	1.83	[1.58, 2.11]	*P* < 0.00001	18	*P =* 0.28
**Research Region**								
	Asia	2	105	2.98	[1.88, 4.73]	*P* < 0.00001	0	*P =* 0.87
	Europe and America	7	950	1.65	[1.41, 1.93]	*P* < 0.00001	0	*P =* 0.73
	Multicentre	2	1,269	1.56	[0.90, 2.69]	*P =* 0.11	87	*P =* 0.005
**Sample Size**								
	> 100	7	2,065	1.6	[1.31, 1.96]	*P* < 0.00001	66	*P =* 0.007
	≤ 100	4	259	2.18	[1.62, 2.93]	*P* < 0.00001	21	*P =* 0.28
**Median/Average Age (Years)**								
	> 60	7	2,058	1.55	[1.28, 1.86]	*P* < 0.00001	60%	*P =* 0.02
	≤ 60	3	210	2.42	[1.76, 3.34]	*P* < 0.00001	0%	*P =* 0.47
**NLR Cutoff**								
	≥ 2	2	596	1.34	[0.99, 1.82]	*P =* 0.06	*I*^2^ = 53%	*P =* 0.15
	≥ 3	6	1,357	1.88	[1.47, 2.39]	*P* < 0.00001	*I*^2^ = 41%	*P =* 0.13
	≥4	3	371	1.83	[1.43, 2.35]	*P* < 0.00001	*I*^2^ = 0%	*P =* 0.47
**Follow-Up Period (Months)**								
	> 12	3	283	1.94	[1.35, 2.79]	*P =* 0.0003	41	*P =* 0.19
	≤ 12	5	937	1.68	[1.25, 2.25]	*P =* 0.0005	72	*P =* 0.007
	NR	3	1,114	1.76	[1.20, 2.58]	*P =* 0.004	55	*P =* 0.11
**Study Quality**								
	> 7	8	1,876	1.84	[1.41, 2.40]	*P* < 0.00001	74	*P =* 0.0003
	≤ 7	3	448	1.56	[1.25, 1.95]	*P* < 0.0001	21	*P* = 0.28
**Publication Year**								
	Before year 2016	4	266	2.41	[1.79, 3.25]	*P* < 0.00001	0	*P =* 0.68
	After year 2016	7	2,058	1.55	[1.28, 1.86]	*P* < 0.00001	60	*P =* 0.02
**Initial Inclusion Period**								
	Before year 2010	8	2,168	1.59	[1.32, 1.91]	*P* < 0.00001	61	*P =* 0.01
	After year 2010	3	161	2.81	[1.88, 4.19]	*P* < 0.00001	0	*P =* 0.87
**Treatment Regimen**								
	Sorafenib	9	1,807	1.69	[1.38, 2.08]	*P* < 0.00001	66	*P =* 0.003
	Combined with Sorafenib	1	40	3.07	[1.70, 5.55]	*P =* 0.0002	–	–
	Tivantinib	1	65	1.58	[1.01, 2.47]	*P =* 0.05	–	–

### PLR and OS

There were only three studies that reported on the relationship between PLR and clinical outcome. However, the analysis showed significant prognostic function for HCC patients with molecular targeted medicine therapy [1.49 (1.16, 1.93), *P* = 0.002, *I*^2^ = 0%, *P* = 0.65, Figure 2B].

### Association Between Other Plasma Inflammation Biomarkers and OS

Several inflammatory biomarkers beside NLR and PLR were also detected in the included studies. In Zhu's study (28), the baseline MLR in 142 HCC patients following sorafenib therapy was significantly related to OS (HR: 0.445, 95% CI: 0.301–0.658, *P* = 0.0001) and PFS (HR: 0.457, 95% CI: 0.308–0.678, *P* = 0.0001). Conroy (24) also showed a significant predictive effect for the LMR [LMR < 3: HR 1.45 (1.02–2.06)] that cannot be combined analyzed with Zhu's data. Yuan's (25) research showed that patients with a lower peripheral neutrophil count have a longer median OS time [11.5 vs. 5.0 months; 1.796 (1.085–2.973), *P* = 0.023]. Miyahara et al. (29) found that high levels of Ang-2, G-CSF, HGF, and leptin may predict shorter progression-free survival with HRs of 2.51, 6.89, 2.55, and 4.14, respectively. Goyal et al. (31) showed that a shorter TTP was associated with higher IL-10 and a lower CD56 Bright NK lymphocyte fraction, higher plasma IL-8, and sMET (*P* < 0.05). Cho et al. (32) reported on 34 advanced HCC patients with HBV history treated with sorafenib and found that a poor PFS correlated with a higher pretreatment serum IL-17A level that was identified as a significant prediction factor (IL-17A > 1.94 pg/mL; HR = 19.96; 95% CI = 3.32–119.86; *P* = 0.001).

### Publication Bias

As shown in Figure S1, the publication bias was analyzed. We did not conduct the funnel plot in the PLR group because only three studies were included. Asymmetric maps in the NLR group showed potential publication bias. However, since there were only 11 studies included, the meaning of the funnel plot may be limited.

## Discussion

Recently, several meta-analysis studies reported the prognostic role of NLR or PLR in HCC patients undergoing liver transplantation or hepatectomy (34–36). Lai et al. analyzed the prognostic effect of PLR on HCC patients following liver transplantation and concluded that high pre-transplant PLR values were connected with an increased risk of post-operative HCC recurrence (7). However, in the study by Zheng et al. (10), the subgroup analysis according to the treatment showed that NLR had no significant prognostic effect on sorafenib in the treatment of liver cancer. It is necessary to reconsider this opinion, however, since there were too few studies involved to draw this conclusion.

As far as we know, there is no specific study on the prognostic effect of NLR and PLR on sorafenib that has been retrieved. Therefore, we conducted this study which is the first meta-analysis of the association between baseline blood NLR and clinical outcomes of advanced HCC patients following sorafenib treatment. In the present research, we comprehensively analyzed 18 studies with a total of 2,745 patients. The pooled result showed that the synthesized HR favored patients with low pretreatment NLR, and also indicated that the HCC patients with a lower pretreatment NLR might have a better response to sorafenib than those with higher NLR [HR = 1.76,95% CI (1.44, 2.15), *P* < 0.00001, *I*^2^ = 68%]. In the research region subgroup, the result of the subgroup analysis indicated that individuals with different genetic backgrounds play a prominent role in the source of heterogeneity, which influenced the significance of heterogeneity in subgroups of sample size, age, follow-up time, and study quality. Interestingly, the predictive effect on OS was more pronounced as the NLR cutoff value increased. When the cutoff value of NLR is 2, the prognosis of the two groups cannot be distinguished, suggesting that 2 is not an optimal cutoff value, while when the cutoff value is set at 3, the prognosis of the two groups can be significantly distinguished, indicating that 3 is the minimum cutoff value for NLR to play a prognostic role. Notably, significant predictive effect of NLR on the clinical outcome was detected in HCC patients treated with sorafenib rather than tivantinib.

Above all, NLR and PLR may be promising and reliable biomarkers for HCC patients and clinical practitioners to make treatment decisions. decisions. Mechanically, elevated neutrophils reflect the response to the system inflammation that related to the increasing tumor burden (37, 38), while lymphocytes induce an antitumor effect and mediating immune function (39). It's reported that 90% of HCCs arise in the context of hepatic injury and inflammation (40). Therefore, NLR could be an easy and effective biomarker that represents systemic inflammation responses to HCC.

In the present study, we also summarized that some blood inflammatory cytokines also have prognostic effects, which are also related to the inflammatory immune response in the process of tumor formation. Tumor development depends to a large extent on various types of immune cells and immunologically active molecules such as programmed death 1/programmed death ligand 1 (PD-1/PD-L1) in the tumor microenvironment (41). IL-6, IL-10, TNF-α (tumor necrosis factor-alpha), and other pro-inflammatory factors mobilize and recruit MDSCs (myeloid-derived suppressor cells), T cells, and macrophages, thereby amplifying the pro-inflammatory response and contributing to the formation of a heterogeneous population of cells. A number of studies have shown that chronic inflammation plays a decisive role in the formation and development of digestive tract tumors, such as *Helicobacter pylori*-associated gastric cancer, colitis-associated colon cancer, hepatitis B, hepatitis C-related liver cancer, etc., providing important evidence for inflammation-induced tumors (42–44). IL-6 has been shown to be involved in the pathogenesis of various tumors (45). MDSC-derived IL-6 can cause dysfunction of CD4+ T cells, reducing the production of interferon gamma (IFN-γ) and resulting in the loss of the ability to attack tumor cells in the subsequent effect phase, which also influences the expression of PD-1/PD-L1 (46, 47). Nagai et al. (48) evaluated the change in cytokines of 57 HCC patients treated by sorafenib, and reported that sorafenib increased the blood TNF-α levels and decreased sFas expression, which might promote TNF- or Fas-related apoptosis at doses ≥400 mg/day. Meanwhile, a significant increase in Teff proliferation was seen in the low dose group with elimination of Treg suppression and increased secretion of IL2 and IL6, which may promote immune responsiveness in HCC patients (49). Therefore, inflammation involved in the formation of HCC may be one of the reasons for its promising prognostic effect. We also attempted to do a subgroup analysis of etiology to detect whether there is a difference of inflammation biomarker in prognosis between viral hepatitis and non-virus etiology. Unfortunately, it cannot be conducted as each study included patients with all kinds of causes.

Although our research implemented strict following of the protocol and inclusion criteria, there are still some limitations. Firstly, the current research mainly analyzed the prognosis effects of NLR and PLR on the OS of advanced HCC patients following treatment with sorafenib, while PFS, RFS, or other clinical outcomes were not evaluated due to the unavailability of data in the included studies. Secondly, in the subgroup analysis, it can be seen that different ethnic groups have a greater impact on the consistency of the research results. This may also be due to the fact that most of the HCC populations in Asia are caused by viral infection, while non-viral background HCC occurs predominantly in Europe and the United States. Thirdly, the sample size was small for the meta-analysis of the prognostic value of PLR on molecular targeted drugs for the treatment of HCC. The included studies were scaled low risk of bias according to the NOS. However, we synthesized the studies and potential publication bias shown in the NLR analysis. Therefore, cautious interpretation is needed. Above all, large-scale prospective studies are encouraged to confirm our findings.

## Conclusion

In conclusion, the present study reported a promising prognostic biomarker for advanced HCC patients following sorafenib treatment, and notably indicated that advanced HCC patients with a lower baseline NLR and PLR may have a better response to sorafenib than those with higher ones. These biomarkers may be explored as one of the assistant tools for clinicians in decision-making of treatment plans. Subgroup analysis showed different genetic backgrounds played a key role in the source of heterogeneity and the prognostic effect was more obvious as the NLR cutoff value increased. Therefore, further large-scale clinical trials to determine the optimal cutoff value for each inflammatory related marker and analysis of their prognostic effectiveness in HCC patients with different infectious virus backgrounds, which is important for identifying the dominant populations for molecular targeted medicine, is encouraged.

## Data Availability Statement

Sufficient data and material are provided in the article and the Supplementary Material.

## Author Contributions

LL and LF gave the initial idea and wrote the manuscript. LL and QZ screened articles and extracted the data. YG and PC analyzed and interpreted the data. All authors read and approved the final manuscript.

### Conflict of Interest

The authors declare that the research was conducted in the absence of any commercial or financial relationships that could be construed as a potential conflict of interest.

## Abbreviations

CI: Confidence Interval
HR: Hazard Ratio
NLR: Neutrophil-to-lymphocyte Ratio
PLR: Platelet-to-lymphocyte Ratio
MLR: Monocyte-to-lymphocyte Ratio
LMR: Lymphocyte-to-Monocyte Ratio
OS: Overall Survival
PFS: Progression Free Survival
RFS: Recurrence Free Survival
TTP: Time to Tumor Progression
TTF: Time to Treatment Failure
TACE: transcatheter arterial chemoembolization therapy
TAE: transcatheter arterial embolization.
