# Supplementary material for: Prognostic Roles of Blood Inflammatory Markers in Hepatocellular Carcinoma Patients Taking Sorafenib. A Systematic Review and Meta-Analysis
Source: Front Oncol. 2020 Jan 29;9:1557. doi: 10.3389/fonc.2019.01557 (PMC7000550; doi:10.3389/fonc.2019.01557)
Supplement: Supplementary file 1 [file Table_1.DOCX]

| Table S1: | | |
| --- | --- | --- |
| Search Strategy Used in PubMed 2018/12/28 | | |
| No. | Search items | Items found |
| [#1](https://www.ncbi.nlm.nih.gov/pubmed/advanced) | Search ((((((((((((((((((Hepatocellular Carcinoma[MeSH Terms]) OR Carcinoma, Hepatocellular) OR Adult Liver Cancer) OR Liver Cancer, Adult) OR Liver Cell Carcinoma, Adult) OR Hepatocellular Carcinomas) OR Carcinomas, Hepatocellular) OR Hepatomas) OR Hepatoma) OR Liver Cell Carcinomas) OR Cell Carcinomas, Liver) OR Cell Carcinoma, Liver) OR Carcinomas, Liver Cell) OR Carcinoma, Liver Cell) OR Liver Cell Carcinoma) OR Liver Cancers, Adult) OR Cancers, Adult Liver) OR Cancer, Adult Liver) OR Adult Liver Cancers Sort by: Best Match | [151526](https://www.ncbi.nlm.nih.gov/pubmed/?cmd=HistorySearch&querykey=22) |
| [#2](https://www.ncbi.nlm.nih.gov/pubmed/advanced) | Search ((((((((((((((((Sorafenib[MeSH Terms]) OR Nexavar) OR BAY 43-9006) OR BAY 43 9006) OR BAY 439006) OR Sorafenib N-Oxide) OR Sorafenib N Oxide) OR BAY-673472) OR BAY 673472) OR BAY 545-9085) OR BAY 545 9085) OR BAY 5459085) OR BAY-545-9085) OR BAY5459085) OR Sorafenib Tosylate)) OR ((((((((Molecular Targeted Therapy[MeSH Terms]) OR Molecular Targeted Therapies) OR Targeted Therapy, Molecular) OR Therapy, Molecular Targeted) OR Targeted Molecular Therapy) OR Molecular Therapy, Targeted) OR Targeted Molecular Therapies) OR Therapy, Targeted Molecular) Sort by: Best Match | [56856](https://www.ncbi.nlm.nih.gov/pubmed/?cmd=HistorySearch&querykey=54) |
| [#3](https://www.ncbi.nlm.nih.gov/pubmed/advanced) | Search ((((((((((cancer-related inflammatory response)[Title/Abstract] OR Inflammatory Markers)[Title/Abstract] OR Neutrophil to Lymphocyte Ratio))[Title/Abstract] OR lymphocyte)[Title/Abstract] OR Neutrophil))[Title/Abstract] OR platelet))[Title/Abstract] OR platelet to Lymphocyte Ratio[Title/Abstract]) Sort by: Best Match | [1048376](https://www.ncbi.nlm.nih.gov/pubmed/?cmd=HistorySearch&querykey=78) |
| [#4](https://www.ncbi.nlm.nih.gov/pubmed/advanced) | Search (((((((((((((cancer-related inflammatory response)[Title/Abstract] OR Inflammatory Markers)[Title/Abstract] OR Neutrophil to Lymphocyte Ratio))[Title/Abstract] OR lymphocyte)[Title/Abstract] OR Neutrophil))[Title/Abstract] OR platelet))[Title/Abstract] OR platelet to Lymphocyte Ratio[Title/Abstract]))) AND (((((((((((((((((Sorafenib[MeSH Terms]) OR Nexavar) OR BAY 43-9006) OR BAY 43 9006) OR BAY 439006) OR Sorafenib N-Oxide) OR Sorafenib N Oxide) OR BAY-673472) OR BAY 673472) OR BAY 545-9085) OR BAY 545 9085) OR BAY 5459085) OR BAY-545-9085) OR BAY5459085) OR Sorafenib Tosylate)) OR ((((((((Molecular Targeted Therapy[MeSH Terms]) OR Molecular Targeted Therapies) OR Targeted Therapy, Molecular) OR Therapy, Molecular Targeted) OR Targeted Molecular Therapy) OR Molecular Therapy, Targeted) OR Targeted Molecular Therapies) OR Therapy, Targeted Molecular))) AND (((((((((((((((((((Hepatocellular Carcinoma[MeSH Terms]) OR Carcinoma, Hepatocellular) OR Adult Liver Cancer) OR Liver Cancer, Adult) OR Liver Cell Carcinoma, Adult) OR Hepatocellular Carcinomas) OR Carcinomas, Hepatocellular) OR Hepatomas) OR Hepatoma) OR Liver Cell Carcinomas) OR Cell Carcinomas, Liver) OR Cell Carcinoma, Liver) OR Carcinomas, Liver Cell) OR Carcinoma, Liver Cell) OR Liver Cell Carcinoma) OR Liver Cancers, Adult) OR Cancers, Adult Liver) OR Cancer, Adult Liver) OR Adult Liver Cancers) Sort by: Best Match | [228](https://www.ncbi.nlm.nih.gov/pubmed/?cmd=HistorySearch&querykey=79) |
