# Supplementary material for: Prognostic Roles of Blood Inflammatory Markers in Hepatocellular Carcinoma Patients Taking Sorafenib. A Systematic Review and Meta-Analysis
Source: Front Oncol. 2020 Jan 29;9:1557. doi: 10.3389/fonc.2019.01557 (PMC7000550; doi:10.3389/fonc.2019.01557)
Supplement: Supplementary file 2 [file Table_2.DOCX]

| Table S2: | | |
| --- | --- | --- |
| Search Strategy Used in Cochrane library 2018/12/28 | | |
| No. | Search items | Items found |
| #1 | MeSH descriptor: [Carcinoma, Hepatocellular] explode all trees | 1482 |
| #2 | (Carcinomas, Hepatocellular):ti,ab,kw | 136 |
| #3 | (Hepatocellular Carcinomas):ti,ab,kw | 136 |
| #4 | (Liver Cell Carcinoma, Adult):ti,ab,kw | 1343 |
| #5 | (Liver Cancer, Adult):ti,ab,kw | 3131 |
| #6 | (Adult Liver Cancer):ti,ab,kw | 3131 |
| #7 | (Adult Liver Cancers):ti,ab,kw | 191 |
| #8 | (Cancer, Adult Liver):ti,ab,kw | 3131 |
| #9 | (Cancers, Adult Liver):ti,ab,kw | 191 |
| #10 | (Liver Cancers, Adult):ti,ab,kw | 191 |
| #11 | (Liver Cell Carcinoma):ti,ab,kw | 2332 |
| #12 | (Carcinoma, Liver Cell):ti,ab,kw | 2332 |
| #13 | (Carcinomas, Liver Cell):ti,ab,kw | 89 |
| #14 | (Cell Carcinoma, Liver):ti,ab,kw | 2332 |
| #15 | (Cell Carcinomas, Liver):ti,ab,kw | 89 |
| #16 | (Liver Cell Carcinomas):ti,ab,kw | 89 |
| #17 | (Hepatocellular Carcinoma):ti,ab,kw | 3403 |
| #18 | (Hepatoma):ti,ab,kw | 111 |
| #19 | (Hepatomas):ti,ab,kw | 10 |
| #20 | #1 OR #2 OR #3 OR #4 OR #5 OR #6 OR #7 OR #8 OR #9 OR #10 OR #11 OR #12 OR #13 OR #14 OR #15 OR #16 OR #17 OR #18 OR #19 | 6364 |
| #21 | MeSH descriptor: [Molecular Targeted Therapy] explode all trees | 114 |
| #22 | (Molecular Targeted Therapies):ti,ab,kw | 219 |
| #23 | (Targeted Therapy, Molecular):ti,ab,kw | 643 |
| #24 | (Therapy, Molecular Targeted):ti,ab,kw | 643 |
| #25 | (Targeted Molecular Therapy):ti,ab,kw | 643 |
| #26 | (Molecular Therapy, Targeted):ti,ab,kw | 643 |
| #27 | (Targeted Molecular Therapies):ti,ab,kw | 219 |
| #28 | (Therapy, Targeted Molecular):ti,ab,kw | 643 |
| #29 | #21 OR #22 OR #23 OR #24 OR #25 OR #26 OR #27 OR #28 | 657 |
| #30 | (Sorafenib ):ti,ab,kw | 1147 |
| #31 | (Targeted Therapy, Molecular):ti,ab,kw | 18 |
| #32 | (Nexavar):ti,ab,kw | 23 |
| #33 | (BAY 43 9006):ti,ab,kw | 23 |
| #34 | (BAY 439006):ti,ab,kw | 0 |
| #35 | (Sorafenib N-Oxide):ti,ab,kw | 0 |
| #36 | (Sorafenib N Oxide):ti,ab,kw | 0 |
| #37 | (BAY-673472 ):ti,ab,kw | 0 |
| #38 | (Sorafenib Tosylate):ti,ab,kw | 18 |
| #39 | #30 OR #31 OR #32 OR #33 OR #34 OR #35 OR #36 OR #37 OR #38 | 1156 |
| #40 | #29 OR #39 | 1761 |
| #41 | (cancer-related inflammatory response):ti,ab,kw | 23 |
| #42 | (Inflammatory Markers):ti,ab,kw | 6000 |
| #43 | (Neutrophil to Lymphocyte Ratio):ti,ab,kw | 334 |
| #44 | (platelet to lymphocyte ratio):ti,ab,kw | 85 |
| #45 | (Neutrophil):ti,ab,kw | 5798 |
| #46 | ( Lymphocyte):ti,ab,kw | 13410 |
| #47 | (platelet):ti,ab,kw | 17159 |
| #48 | #41 OR #42 OR #43 OR #44 OR #45 OR #46 OR #47 | 39473 |
| #49 | #20 AND #40 AND #48 | 46 |
