# Supplementary material for: Prognostic Roles of Blood Inflammatory Markers in Hepatocellular Carcinoma Patients Taking Sorafenib. A Systematic Review and Meta-Analysis
Source: Front Oncol. 2020 Jan 29;9:1557. doi: 10.3389/fonc.2019.01557 (PMC7000550; doi:10.3389/fonc.2019.01557)
Supplement: Supplementary file 3 [file Table_3.DOCX]

| Table S3: | | |
| --- | --- | --- |
| Search Strategy Used in Embase 2018/12/28 | | |
| No. | Search items | Items found |
| #1 | 'carcinoma, hepatocellular'/exp OR 'carcinoma,hepatocellular' OR (carcinoma, AND hepatocellular) | 160,076 |
| #2 | carcinomas, hepatocellular' OR (carcinomas, AND hepatocellular) | 8,537 |
| #3 | hepatocellular carcinomas' OR (hepatocellular AND carcinomas) | 8,537 |
| #4 | liver cell carcinoma, adult' OR (('liver'/exp OR liver) AND ('cell'/exp OR cell) AND carcinoma, AND ('adult'/exp OR adult)) | 62,688 |
| #5 | liver cancer, adult' OR (('liver'/exp OR liver)AND cancer, AND ('adult'/exp OR adult)) | 114,386 |
| #6 | adult liver cancer' OR (('adult'/exp OR adult)AND ('liver'/exp OR liver) AND ('cancer'/exp OR cancer)) | 150,544 |
| #7 | adult liver cancers' OR (('adult'/exp OR adult) AND ('liver'/exp OR liver) AND ('cancers'/exp OR cancers)) | 134,997 |
| #8 | cancer, adult liver' OR (cancer, AND ('adult'/exp OR adult) AND ('liver'/exp OR liver)) | 114,386 |
| #9 | cancers, adult liver' OR (cancers, AND ('adult'/exp OR adult) AND ('liver'/exp OR liver)) | 9,768 |
| #10 | liver cancers, adult' OR (('liver'/exp OR liver)AND cancers, AND ('adult'/exp OR adult)) | 9,768 |
| #11 | liver cell carcinoma'/exp OR 'liver cell carcinoma' OR (('liver'/exp OR liver) AND ('cell'/exp OR cell) AND ('carcinoma'/exp OR carcinoma)) | 184,319 |
| #12 | carcinoma, liver cell'/exp OR 'carcinoma, liver cell' OR (carcinoma, AND ('liver'/exp OR liver) AND ('cell'/exp OR cell)) | 177,223 |
| #13 | carcinomas, liver cell' OR (carcinomas, AND('liver'/exp OR liver) AND ('cell'/exp OR cell)) | 11,815 |
| #14 | cell carcinoma, liver' OR (('cell'/exp OR cell)AND carcinoma, AND ('liver'/exp OR liver)) | 177,182 |
| #15 | cell carcinomas, liver' OR (('cell'/exp OR cell)AND carcinomas, AND ('liver'/exp OR liver)) | 11,815 |
| #16 | liver cell carcinomas' OR (('liver'/exp OR liver) AND ('cell'/exp OR cell) AND carcinomas) | 11,815 |
| #17 | hepatocellular carcinoma'/exp OR 'hepatocellular carcinoma' OR (hepatocellular AND ('carcinoma'/exp OR carcinoma)) | 160,176 |
| #18 | 'hepatoma'/exp OR hepatoma | 165,195 |
| #19 | hepatomas | 2,679 |
| #20 | #1 OR #2 OR #3 OR #4 OR #5 OR #6 OR #7 OR #8 OR #9 OR #10 OR #11 OR #12 OR #13 OR #14 OR #15 OR #16 OR #17 OR #18 OR #19 | 300,576 |
| #21 | 'molecular targeted therapy'/exp OR 'molecular targeted therapy' OR (molecular AND targeted AND ('therapy'/exp OR therapy)) | 78,355 |
| #22 | molecular targeted therapies' OR (molecular AND targeted AND therapies) | 21,749 |
| #23 | targeted therapy, molecular' OR (targeted AND therapy, AND molecular) | 57,809 |
| #24 | therapy, molecular targeted' OR (therapy, AND molecular AND targeted) | 57,809 |
| #25 | targeted molecular therapy'/exp OR 'targeted molecular therapy' OR (targeted AND molecular AND ('therapy'/exp OR therapy)) | 78,355 |
| #26 | molecular therapy, targeted' OR (molecular AND therapy, AND targeted) | 57,809 |
| #27 | targeted molecular therapies' OR (targeted AND molecular AND therapies) | 21,749 |
| #28 | therapy, targeted molecular' OR (therapy, AND targeted AND molecular) | 57,809 |
| #29 | 'sorafenib'/exp OR sorafenib | 26,197 |
| #30 | 'nexavar'/exp OR nexavar | 25,427 |
| #31 | 'bay 43-9006'/exp OR 'bay 43-9006' OR (('bay'/exp OR bay) AND '43 9006') | 25,423 |
| #32 | bay 43 9006'/exp OR 'bay 43 9006' OR (('bay'/exp OR bay) AND 43 AND 9006) | 25,426 |
| #33 | bay 439006'/exp OR 'bay 439006' OR (('bay'/exp OR bay) AND 439006) | 25,412 |
| #34 | 'sorafenib n-oxide' OR (('sorafenib'/exp OR sorafenib) AND ('n oxide'/exp OR 'n oxide')) | 50 |
| #35 | 'sorafenib n oxide' OR (('sorafenib'/exp OR sorafenib) AND n AND ('oxide'/exp OR oxide)) | 233 |
| #36 | sorafenib tosylate'/exp OR 'sorafenib tosylate'OR (('sorafenib'/exp OR sorafenib) AND tosylate) | 25,418 |
| #37 | #21 OR #22 OR #23 OR #24 OR #25 OR #26 OR #27 OR #28 OR #29 OR #30 OR #31 OR #32 OR #33 OR #34 OR #35 OR #36 | 103,511 |
| #38 | cancer-related inflammatory response' OR ('cancer related' AND inflammatory AND ('response'/exp OR response)) | 473 |
| #39 | inflammatory markers' OR (inflammatory AND markers) | 75,841 |
| #40 | neutrophil to lymphocyte ratio':ti,ab,kw | 3,731 |
| #41 | 'platelet to lymphocyte ratio':ti,ab,kw | 1,073 |
| #42 | #38 OR #39 OR #40 OR #41 | 79,824 |
| #43 | #20 AND #37 AND #42 | 68 |
