# Supplementary material for: Prognostic Roles of Blood Inflammatory Markers in Hepatocellular Carcinoma Patients Taking Sorafenib. A Systematic Review and Meta-Analysis
Source: Front Oncol. 2020 Jan 29;9:1557. doi: 10.3389/fonc.2019.01557 (PMC7000550; doi:10.3389/fonc.2019.01557)
Supplement: Supplementary file 4 [file Table_4.DOCX]

| Table S4: | | |
| --- | --- | --- |
| Search Strategy Used in Web of Science 2018/12/28 | | |
| No. | Search items | Items found |
| #1 | (Hepatocellular Carcinoma) OR (Carcinoma, Hepatocellular) OR (Adult Liver Cancer) OR (Liver Cancer, Adult) OR (Liver Cell Carcinoma, Adult) OR (Hepatocellular Carcinomas) OR (Carcinomas, Hepatocellular) OR (Hepatomas) OR (Hepatoma) OR (Liver Cell Carcinomas) OR (Cell Carcinomas, Liver) OR (Cell Carcinoma, Liver) OR (Carcinomas, Liver Cell) OR (Carcinoma, Liver Cell) OR (Liver Cell Carcinoma) OR (Liver Cancers, Adult) OR (Cancers, Adult Liver) OR (Cancer, Adult Liver) OR (Adult Liver Cancers) | 280300 |
| #2 | (Molecular Targeted Therapy) OR (Molecular Targeted Therapies) OR (Targeted Therapy, Molecular) OR (Therapy, Molecular Targeted) OR (Targeted Molecular Therapy) OR (Molecular Therapy, Targeted) OR (Targeted Molecular Therapies) OR (Therapy, Targeted Molecular) | 326210 |
| #3 | (Sorafenib) OR (Nexavar) OR (BAY 43-9006) OR (BAY 43 9006) OR (BAY 439006) OR (Sorafenib N-Oxide) OR (Sorafenib N Oxide) OR (BAY-673472) OR (BAY 673472) OR (BAY 545-9085) OR (BAY 545 9085) OR (BAY 5459085) OR (BAY-545-9085) OR (BAY5459085) OR (Sorafenib Tosylate) | 14777 |
| #4 | #2 or #3 | 337960 |
| #5 | (cancer-related inflammatory response) OR (Inflammatory Markers) OR (Neutrophil to Lymphocyte Ratio) OR (platelet to lymphocyte ratio) | 102235 |
| #6 | #1 AND #4 AND #5 | 128 |
