# Supplementary material for: Prognostic Roles of Blood Inflammatory Markers in Hepatocellular Carcinoma Patients Taking Sorafenib. A Systematic Review and Meta-Analysis
Source: Front Oncol. 2020 Jan 29;9:1557. doi: 10.3389/fonc.2019.01557 (PMC7000550; doi:10.3389/fonc.2019.01557)
Supplement: Supplementary file 5 [file Table_5.DOCX]

| Table S5 | |
| --- | --- |
| Characteristics of Excluded Studies | |
| Study | Reason for exclusion |
| Agilli 2014 | This article is a comment. |
| Li 2014 | The HCC patients in this study were not received molecular-targeted therapy. |
| Luè 2014 | It's a conference abstract and there is a related full text publication authored by Luè.(Luè 2017) |
| Peng 2014 | The patients with small HCC who underwent curative resection were included rather than molecular-targeted therapy. |
| Tanoglu 2014 | This article is a comment. |
| Sukato 2015 | The HCC patients in this study were treated with radioembolization rather than molecular-targeted therapy. |
| Tajiri 2015 | The HCC patients in this study were treated with arterial cisplatin plus continuous 5-fluorouracil rather than molecular-targeted therapy. |
| Terashima 2015 | The HCC patients in this study were treated with hepatic arterial infusion chemotherapy (HAIC) ratrher than molecular-targeted therapy. |
| Obi 2016 | It's a conference abstract without sufficient data. |
| Son 2017 | The HCC patients included in this study were treated with radiotherapy rather thanmolecular-targeted therapy. |
| Taussig 2017 | The HCC patients included in this study were treated with intra-arterial therapy (chemoembolization or radioembolization) rather than molecular-targeted therapy. |
| Shen 2016 | The HCC patients included in this study were treated with hepatectomy. |
| Li 2015 | The HCC patients in this study were not received molecular-targeted therapy. |
| Reig 2016 | It's a conference abstract without sufficient data. |
| Wang 2017 | In this article, 33.7% patients underwent chemotherapy with sorafenib and 11.5% patients underwent curative treatment including surgical resection, radio-frequency ablation or liver transplant. However, we only focus on the patients treated with molecular-targeted therapy. |
| Sprinzl 2018 | It's a conference abstract and the data is not available. |
| Negri 2011 | It's a conference abstract and the study is not related with our research. |
| Lee 2014 | It's a conference abstract without sufficient data. |
| Zhu 2014 | It's a conference abstract and the study is not related with our research. |
| Nagai 2015 | The study is not related with our research. |
| Xue 2015 | The study is not related with our research. |
| Howell 2016 | It's a conference abstract and there is a related full text publication authored by Howell.(Howell 2017) |
| Pinato 2016 | It's a conference abstract and the data is not available. |
| Shiozawa 2016 | Article in Japanese |
| Sun 2016 | The study is not related with our research |
| Yada 2016 | The study is not related with our research. |
| Sprinzl 2017 | It's a conference abstract and the data is not available. |
| Tsunematsu 2017 | The HCC patients in this study were treated with hepatic arterial infusion chemotherapy (HAIC) ratrher than molecular-targeted therapy. |
| Kim 2018 | In this article, a risk scoring system was developed with six covariates: etiology, platelet count, Barcelona Clinic Liver Cancer stage, protein induced by vitamin K absence-II, HGF, and FGF.However, it's not related with our research. |
| Li 2018 | It's a conference abstract without sufficient data. |
| Llovet 2008 | The results in this study are not the related inflammation biomarkers which we focused. |
| Scanga 2009 | It's a comment article. |
| Song 2009 | It's a review article. |
| Greten 2010 | The study is about evaluating safety and efficacy of a telomerase peptide vaccination which is not related with our research. |
| Nagai 2012 | The study result is not related with our research |
| Singhal 2012 | It's a review article about molecular and serum markers in hepatocellular carcinoma. |
| Yeon 2012 | It's a conference abstract and the study is not related with our research. |
| Berk 2013 | The study result is not related with our research |
| Cabrera 2013 | This study researched the sorafenib function on immune modulation of effector CD4+ and regulatory T cell in patients with hepatocellular carcinoma,which did not report the relationship with overall survival. |
| Cainap 2013 | The study result is not related with our research |
| Nagai 2014 | This article studied the changes in blood cytokines after the sorafenib treatment but did not report the relationship with the overall survival. |
| Chao 2015 | The study result is not related with our research |
| Cheng 2015 | It's a conference abstract and the study is not related with our research. |
| Cheng 2016 | The study analyzed the vascular endothelial growth factor receptor 1 (sVEGFR1) and (B) hepatocyte growth factor (HGF) as the pronostic biomarker of dovitinib and sorafenib.Howerver, we try to find the blood inflammation biomaker for molecular-targeted medicne. |
| Lee 2016 | The study result is not related with our research |
| Zhou 2016 | The study result is not related with our research |
| El-Khoueiry 2017 | The study is not related with our research |
| Han 2017 | The study result is not related with our research |
| Kelley 2017 | The study result is not related with our research |
| Kudo 2017 | The study result is not related with our research |
| Pan 2017 | The study is not related with our research |
| Patt 2017 | The study result is not related with our research |
| Choo 2018 | It's a conference abstract and the study is not related with our research. |
| Furuse 2018 | It's a conference abstract and the study is not related with our research. |
| Ikeda 2018 | The study result is not related with our research |
| Jordan 2018 | It's a conference abstract and the included patients were treated with radioembolization. |
| Koral 2018 | The study is animal experiment and not related with our research |
| Kudo 2018 | The study result is not related with our research |
| Kudo 2018 | The study result is not related with our research |
| Reiss 2018 | It's a conference abstract and the study is not related with our research. |
| Tak 2018 | The study result is not related with our research |
| Yen 2018 | The study result is not related with our research |
| Gao 2015 | The included patients were treated with not only molecular-targeted therapy but also chemo or TAE. |
| Diaz-Beveridge 2018 | Duplicated article. |
