# Supplementary material for: Prognostic Roles of Blood Inflammatory Markers in Hepatocellular Carcinoma Patients Taking Sorafenib. A Systematic Review and Meta-Analysis
Source: Front Oncol. 2020 Jan 29;9:1557. doi: 10.3389/fonc.2019.01557 (PMC7000550; doi:10.3389/fonc.2019.01557)
Supplement: Supplementary file 6 [file Table_6.DOCX]

| Table S6 | | | | | | | | | | |
| --- | --- | --- | --- | --- | --- | --- | --- | --- | --- | --- |
| NEWCASTLE - OTTAWA QUALITY ASSESSMENT | | | | | | | | | | |
| Studies | Selection | | | | Comparability | | Outcomes | | | Quality score |
|  | Representativeness of the exposed cohort | Selection the non exposed cohort | Ascertainment of exposure | Demonstration that outcome of interest was not present at start of study | Study controls for factor: like age. | Study controls for any additional factor | Assessment of outcomes | Follow-up long enough | Adequacy of follow up of cohorts |  |
| Zheng 2013 | Yes | Yes | Yes | Yes | Yes | Yes | Yes | Yes | Yes | 9 |
| Fonseca 2014 | Yes | Yes | Yes | Yes | NR | Yes | Yes | Yes | Yes | 8 |
| Wei 2014 | Yes | Yes | Yes | Yes | Yes | Yes | Yes | Yes | Yes | 9 |
| Diaz-Beveridge 2018 | Yes | Yes | Yes | Yes | NR | Yes | Yes | Yes | Yes | 8 |
| Zhang 2015 | Yes | Yes | Yes | Yes | Yes | Yes | Yes | Yes | Yes | 9 |
| Luè 2017 | Yes | Yes | Yes | Yes | NR | Yes | Yes | Yes | Yes | 8 |
| Personeni 2017 | Yes | Yes | Yes | Yes | NR | Yes | Yes | Yes | Yes | 8 |
| Yuan 2017 | Yes | Yes | Yes | Yes | Yes | Yes | Yes | Yes | Yes | 9 |
| Bruix 2017 | Yes | Yes | Yes | Yes | Yes | Yes | Yes | Yes | Yes | 9 |
| Howell 2017 | Yes | Yes | Yes | Yes | Yes | Yes | Yes | Yes | Yes | 9 |
| Afshar 2018 | Yes | Yes | Yes | Yes | Yes | Yes | Yes | NR | NR | 7 |
| Zhu 2018 | Yes | Yes | Yes | Yes | Yes | Yes | Yes | Yes | NR | 8 |
| Casadei Gardini 2016 | Yes | Yes | Yes | Yes | Yes | Yes | Yes | NR | NR | 7 |
| Miyahara 2011 | Yes | Yes | Yes | Yes | Yes | Yes | Yes | Yes | Yes | 9 |
| Katayama 2018 | No | Yes | Yes | Yes | Yes | Yes | Yes | Yes | Yes | 8 |
| Goyal 2018 | Yes | Yes | Yes | Yes | NR | Yes | Yes | Yes | Yes | 8 |
| Cho 2017 | No | Yes | Yes | Yes | Yes | Yes | Yes | Yes | Yes | 8 |
| Conroy 2017 | Yes | Yes | Yes | Yes | NR | NR | Yes | Yes | Yes | 7 |
