# Supplementary figures and images for: Prognostic Roles of Blood Inflammatory Markers in Hepatocellular Carcinoma Patients Taking Sorafenib. A Systematic Review and Meta-Analysis
Source: Front Oncol. 2020 Jan 29;9:1557. doi: 10.3389/fonc.2019.01557 (PMC7000550; doi:10.3389/fonc.2019.01557)

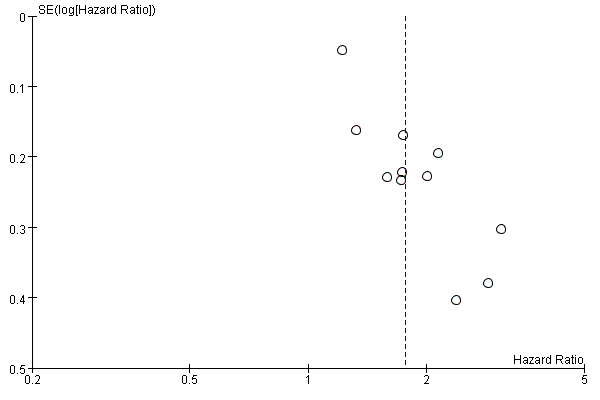

Supplement: Supplementary file 7 [file Image_1.PNG]
